# Walnut oil improves spatial memory in rats and increases the expression of acid‐sensing ion channel genes Asic2a and Asic4

**DOI:** 10.1002/fsn3.889

**Published:** 2018-11-28

**Authors:** Li‐Mei Wang, Yang Yi, Yi‐Lan Yao, Ge Feng, Chang Shu, Hong‐Xun Wang, Xi‐Feng Zhang

**Affiliations:** ^1^ College of Biological and Pharmaceutical Engineering Wuhan Polytechnic University Wuhan China; ^2^ Hubei Key Laboratory for Processing and Transformation of Agricultural Products Wuhan Polytechnic University Wuhan China; ^3^ Key Laboratory for Deep Processing of Major Grain and Oil(Wuhan Polytechnic University)of Ministry of Education in China Wuhan China; ^4^ College of Food Science and Engineering Wuhan Polytechnic University Wuhan China

**Keywords:** acid‐sensing ion channels, Asic2a, Asic4, methylation, morris water maze, walnut oil

## Abstract

Although Walnut oil (WO) has been reported to enhance cognitive function, the underlying molecular mechanisms are not well understood. This study was designed to assess the effects of WO on spatial memory in rats through modulation of the expression of acid‐sensing ion channel genes, *Asic2a* and *Asic4*. To investigate the effect of WO on cognitive performance, we supplemented the diet of female rats with WO. The results showed that supplementation with WO at doses of 2.2 and 11 g kg^−1^ day^−1^ significantly improved learning and memory. In vitro treatment of rat hippocampal neuronal cells with appropriate doses of WO revealed a significant increase in the expression of *Asic2a* and *Asic*4 in a dose‐dependent manner at both the mRNA and protein levels. We conclude that WO intake might help to prevent cognitive decline, particularly in the elderly, and that ASIC genes in neurons can be the targets of compounds contained in the oil.

## 1 INTRODUCTION

Walnut oil (WO) is a highly nutritious edible oil that is rich in monounsaturated fatty acids, polyunsaturated fatty acids (PUFAs), lecithin, and vitamin E, all of which are essential for human health. The proportions of linoleic acid and a‐linolenic acid (50.15%–51.36% and 10.48%–12.04%, respectively) are higher in WO than in other edible oils (Gharibzahedi, Mousavi, Hamedi, & Khodaiyan, 2014; Iwamoto et al., 2002; Zhang et al., 2011). Monounsaturated and polyunsaturated fatty acids can decrease the levels of cholesterol, triglyceride, and low‐density lipoprotein in the blood, and adequate dietary intake may protect against atherosclerosis, coronary heart disease, and hypertension (De and Salen, 2004; Kim, Yokoyama, & Davis, 2014). Phospholipids in WO have also been shown to have a beneficial effect on the brain (Bourre, 2005). Walnuts contain arginine and oleic acid, both have antioxidant properties and may protect against coronary heart disease, stroke, and Alzheimer's disease. WO has a number of health benefits, including reduction in inflammation, improvement of blood circulation, reduction in the risk of heart disease, anti‐aging properties, prevention of eczema, and stabilization of body hormones (Anand & Kaithwas, 2014; Berryman et al., 2013; Müllner et al., 2013). WO can diminish oxidative stress because of its antioxidant properties (Laubertová, Koňariková, Gbelcová, Ďuračková, & Žitňanová, 2015). Walnut polyphenols have superoxide dismutase (SOD)‐like activity and have a remarkable radical scavenging effect against 1,1‐diphenyl‐2‐picrylhydrazyl (Fukuda, Ito, & Yoshida, 2003).

### 1.1 Rationale

Acid‐sensing ion channels (ASICs), which are located in the central and peripheral nervous systems, are proton‐gated ion channels that belong to the DEG/EnaC superfamily of amiloride‐sensitive Na^+^ channels (Waldmann, 2001; Waldmann & Lazdunski, 1998). Six different ASICs (ASIC1a, ASIC1b, ASIC2a, ASIC2b, ASIC3, and ASIC4), encoded by four genes, have been cloned and shown to assemble into multimeric channels (Benson et al., 2002; Jasti, Furukawa, Gonzales, & Gouaux, 2007; Waldmann, 2001; Waldmann & Lazdunski, 1998). Almost all ASIC subunits are expressed in primary afferent sensory neurons, and the subunit composition of each channel determines its unique biophysical and pharmacological properties (Benson et al., 2002; Jasti et al., 2007). ASICs in central neurons are mainly composed of homomeric ASIC1a and heteromeric ASIC1a/2b. Protons and ASICs form a neurotransmitter receptor pair that is essential for acid‐activated currents in processes underlying synaptic plasticity, learning, and memory (Du et al., 2014; Wemmie et al., 2002). Most ASIC subunits (including ASIC2a) are activated by protons, but ASIC2b (a splice variant of ASIC2a) is acid‐insensitive. ASIC1a, activated by acidity in the physiological range (pH> 5.0), plays a role in the formation of learning and memory and is particularly important for innate fear responses and acquired fear‐conditioned behaviors (Coryell et al., 2008; Wemmie et al., 2003; Xiong et al., 2004). ASIC2, like ASIC1, plays a key role in determining the defensive response to aversive stimuli and is important for normal brain function (Price et al., 2014). ASIC2 has also been detected in cutaneous rapidly adapting low‐threshold mechanoreceptors, suggesting a role in the sense of touch. In particular, transcripts for ASIC2a have been detected predominantly in the brain (Fukuda et al., 2003; Laubertová et al., 2015). ASIC2b, a modulatory subunit, does not form a functional channel; instead, it alters the properties of the other subunits (Vila‐Carriles et al., 2006). Genetic deletion of *Asic2a* or *Asic2b* in mice leads to neurological behavior deficits (Cabo et al., 2012; Price et al., 2014). ASIC2a and ASIC3 are the major ASIC subunits in cardiac dorsal root ganglia neurons and provide potential molecular targets to attenuate chest pain and deleterious reflexes associated with cardiac diseases (Hattori et al., 2009). ASIC3 is predominantly expressed in peripheral sensory neurons and contributes to pain sensations, including those from the heart and skeletal muscle (Deval et al., 2008). ASIC4 is broadly expressed in the mammalian nervous system but has no known functions (Donier, Rugiero, Jacob, & Wood, 2008).

In this study, we investigated the effects of WO on rat hippocampal‐dependent cognitive performance and explored the impacts of WO on the expression of *Asic2a* and *Asic4* genes in hippocampal neurons in vitro.

## 2 MATERIALS AND METHODS

### 2.1 Animals

Female Sprague Dawley rats were provided by the Institute of Poultry Science of the Chinese Academy of Agriculture Sciences. Procedures involving the care and use of animals conformed to the U.S. National Institutes of Health guidelines (NIH Pub. No. 85–23, revised 1996) and were approved by the Ethical Committee of Wuhan Polytechnic University. Rats were housed under temperature‐controlled (21–22°C) and light‐controlled (12‐hr light, 12‐hr dark cycle) conditions.

### 2.2 Treatment

Walnut oil was purchased from Hubei Lishizhen Health Care Oil Co., Ltd (Wuhan, China). Walnuts contain around 5.3% hexadecanoic acid, 1.72% octadecanoic acid, 19.25% 9‐octadecenoic acid, 53.83% 9,12‐octadecadienoic acid, and 17.79% 9,12,15‐octadecatrienoic acid.

Walnut oil was orally administered to prepubertal rats (4 weeks old, 100 rats) at three different doses (1.1, 2.2, 11 g/kg) for 30 days. WO was diluted in 0.5% sodium carboxymethyl cellulose solution. The control group was given an equal volume of 0.5% sodium carboxymethyl cellulose solution.

### 2.3 Morris water maze (MWM) test

The hippocampal‐dependent spatial learning capability of rats was evaluated using the MMW test by following the protocol described previously (Pan et al., 2011). The Morris water maze was a black circular pool with a diameter of 150 cm and a height of 80 cm. The temperature of water was 21 to 22°C, the pool was filled to a height of 50 cm, and the maze was geographically divided into four equal quadrants. The rats were subjected to daily sessions of four trials for 5 days. In the tests, the rats had to find the submerged platform that was located in the center of a quadrant of the tank; the submerged platform remained at the same position throughout the training. A video camera was mounted above the center of the tank, and all trials were recorded. The rat was placed in the water facing the wall at one of four random start locations (north, south, east, and west). Each rat was then allowed to find the submerged platform within 60 s and to rest on it for 10 s. If the rat failed to find the hidden platform within the assigned time, it was placed on the platform for 10 s. The rat was then returned to its heated cage for a 30‐s inter‐trial interval. The escape latency (time to reach the platform) was used as an index of performance in this task. One day after the last training session, a probe trial was performed for 60 s, in which the platform was removed from the tank. The rat was placed in the water at the same random start location. The time spent in the quadrant of the former platform position and the correct annulus crossings, that is, the number of times the animal passed through the circular area (diameter, 10 cm) that formerly contained the submerged platform during acquisition, were taken as measures of spatial memory.

### 2.4 Measurements of AChE, NOS, and SOD activity and MDA levels

After the MWM, all rats were sacrificed by a combination of carbon dioxide and cervical dislocation. The whole brain was rapidly dissected (cerebellum discarded) and individually homogenized in cold saline at 10% v/v. After centrifugation at 28,360 *g* for 10 min, the supernatant was collected and used for analyses of acetylcholinesterase (AChE), superoxide dismutase (SOD), NO synthase (NOS), and malondialdehyde (MDA), an index of lipid peroxidation. All measurements were performed using corresponding commercial kits purchased from Nanjing Jiancheng Bioengineering Institute Co., Ltd (Nanjing, China) according to the manufacturer's instructions.

### 2.5 Culturing of hippocampal neurons

The hippocampi of neonatal (day 1) female rats were dissected aseptically under a microscope and, after removal of the meninges, triturated in a serum‐free neurobasal culture medium. Tissues were incubated in trypsin (0.25%) for 10 min at 37°C, and individual cells were dissociated by triturating the tissue with a fire‐polished glass pipette. Cells were spun down, resuspended in culture medium (89% neurobasal + 10% fetal bovine serum + 1% penicillin, and streptomycin) and plated at a density of 10^6^ cells per flask. After 24 hr, the medium was changed to FBS‐free medium (neurobasal/B27 supplemented with 0.5 mM L‐glutamine, penicillin, and streptomycin) (Hidekazu, 2002). WO was added to this medium at final concentrations of 3, 10, 30, 60, and 100 μg/ml on day 4 of the culture. The samples were analyzed on day 6.

### 2.6 RNA isolation and quantitative RT‐PCR

Total RNA was isolated from neuronal cells using an RNA Prep Pure Micro Kit (Tiangen, Beijing, China), according to the manufacturer's instructions. cDNA was synthesized using a PrimeScript^™^ RT Reagent Kit (TaKaRa, Dalian, China), according to the manufacturer's instructions. The reaction system contained 25 pmol oligo‐dT primer and 50 pmol random primers. The RNA was added, and the final volume adjusted to 20 μl using 5× PrimerScript^™^ buffer. These experiments were carried out to quantify gene expression using a SYBR Premix Ex TaqTM Kit (TaKaRa) and an ABI 7300 real‐time PCR instrument (Applied Biosystems, Foster City, CA, USA). Relative RNA equivalents for each sample were obtained using β‐actin as a standard. The sequences of gene primer sets are provided in Table 1.

**Table 1:** Primers used for amplification of specific genes by real‐time PCR

Gene	Primer sequence
*ASIC2a*	F: 5′‐TGACATTGGTGGTCAAATGG ‐3′
	F: 5′‐CTGATGGTTTCGGAGTGGTT ‐3′
*ASIC4*	F: 5′‐GGGAGAACTTCTTGGTCCTG ‐3′
	F: 5′‐CGATCCCAGGAGACCTCATA ‐3′
β*‐actin*	F: 5′‐CCTTGTTCTCAAAAGAGATGTTGAA ‐3′
	R: 5′‐TGACCAGAAACTGGAGAAGATGATA ‐3′

### 2.7 DNA isolation and bisulfite sequencing

DNA was extracted from neuronal cells using a TIANamp Micro DNA Kit (Tiangen, Beijing, China), according to the manufacturer's instructions. DNA was then treated with sodium bisulfite supplied with a Methylamp™ DNA modification kit (Epigentek, Los Angeles, CA, USA), according to the manufacturer's instructions. The bisulfite‐treated DNA was amplified by nested (semi‐nested) PCR for *ASIC2a* and *ASIC4* genes using the primers provided in Table 2. The PCR products were separated by electrophoresis in 1% agarose gel. The correct bands were excised from the gel and purified using a Wizard^®^ SV Gel and PCR Clean‐Up System (Promega, Madison, WI, USA). The purified DNA was then cloned into a pDM19‐T Vector (TaKaRa), according to the manufacturer's instructions. The positive clones were obtained by antibiotic selection, and the insert was sequenced by Invitrogen (Shanghai, China).

**Table 2:** Primers used for amplification of ASIC2a and ASIC4 for methylation analysis

Gene	Primer sequence	Amplified fragment length (bp)
ASIC2a	F: 5′‐GGGTATAGAGTTTAGGGATTGGAG‐3′	296
	R: 5′‐CAACAACAATAAAAACAACAACAAC ‐3′	
ASIC4	F: 5′‐ GTGTTGATAAGGGAAGTTATA ‐3′	347
	R: 5′‐ CTACCTCCTTCTCCTTAAATTT ‐3′	

### 2.8 Western blot

Proteins were extracted from neuronal cells using RIPA lysis solution (Beyotime, Shanghai, China). Western blot analyses were performed according to standard procedures. Briefly, following 10% sodium dodecyl sulfate–polyacrylamide gel electrophoresis, proteins were transferred to polyvinylidene fluoride membranes, blocked with Tris‐buffered saline Tween‐20 (TBST) containing 10% BSA and incubated with the specific primary antibodies, anti‐actin (1.0 μg/ml; Abcam, Hong Kong, China) and either anti‐ASIC2a (0.5 μg/ml, ab77384, Abcam) or anti‐ASIC4 (0.5 μg/ml; ab65700, Abcam). After incubation with secondary antibodies (Beyotime), the blots were developed by enhanced chemiluminescence (Beyotime). Western blot analyses of ASIC2a were performed as previously described (Miao, Zhang, Lin, Lu, & Qiu, 2010). Band intensity was quantified using β‐actin as an internal standard and measured using ImageJ software (Wayne Rasband, National Institutes of Health, Bethesda, Maryland, USA).

### 2.9 Statistical methods

All measurements were repeated at least three times, and data are reported as mean ±*SD*. Data were analyzed using a *t* test or one‐way analysis of variance (ANOVA), followed by the Tukey test for multiple comparisons to determine statistical difference between groups (denoted by an asterisk or different letters), using GraphPad Prism analysis software (San Diego, CA, USA). *p*‐values of less than 0.05 were considered to be statistically significant.

## 3 RESULTS AND DISCUSSION

### 3.1 Walnut oil improves hippocampal‐dependent cognitive performance

**Figure 1:**
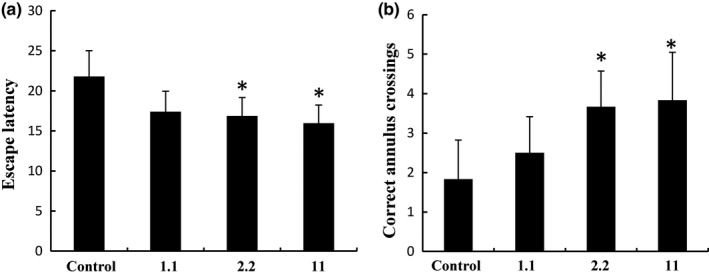
Latency in the Morris water maze hidden platform task, target quadrant time ratios, and correct number of annulus crossings. (a) Comparison of latency times between control, 1.1, 2.2, and 11 g kg^−1^ day^−1^ groups. (b) Correct number of annulus crossings between the control group and the walnut oil‐treated groups in the probe trials of the Morris water maze task. Data are presented as mean±*SD*. *p < 0.05, compared with the control group

Spatial learning and memory performance were evaluated using the MWM test. There was no statistical difference for swimming speed between the control group and the treatment groups receiving 1.1, 2.2, and 11 g/kg WO per day, indicating that the baseline swimming ability of the rats was similar in all groups (data not shown). Escape latencies were decreased over time and were significantly lower in rats treated with 2.2 and 11 g/kg WO compared with that in control group by the fifth day of the acquisition phase (*n* = 10, *p* < 0.05, Figure 1a). Moreover, the latency in the 11 g/kg group was lower than that in the 2.2 g/kg group, indicating a dose‐dependent effect. The probe trial was carried out on the sixth day. Rats in the groups treated with 2.2 and 11 g/kg WO had more annulus crossings in the target quadrant than that in the control group (*n* = 10, *p* < 0.05; Figure 1b). The number of correct annulus crossings was significantly greater in the 11 g/kg group than that in the 2.2 g/kg group, further indicating a dose‐dependent effect.

Our results indicated that in vivo dietary supplementation with WO improved spatial memory. The hidden platform was faster in WO treatment groups than that in the control group by MWM test, indicating that WO improved the spatial memory. We therefore conclude that WO might exert on learning and memory in vivo.

Walnuts are a natural, organic food with a high nutritional value for the brain. WO refined from good‐quality walnuts contains around 50% of the ω‐3 unsaturated fatty acid and linoleic acid. Linoleic acid is a precursor of eicosapentaenoic acid (EPA) and docosahexaenoic acid (DHA) and is essential in human nutrition (Rossignoli et al., 2018). WO has been shown to have a number of health benefits. Numerous experiments have demonstrated that ω‐3 fatty acids can enhance learning and memory. The linolenic acid in WO may contribute to the synthesis of DHA and EPA in the body (Goyens, Spilker, Zock, Katan, & Mensink, 2006). Long‐chain n‐3 PUFAs, particularly EPA and DHA, play a key role in the maintenance of brain functions such as learning and memory which are impaired during aging (De Souza, Fernandes, & do Carmo, 2011). A protective role has been proposed for w‐3 fatty acids in mild cognitive impairment, dementia, and Alzheimer's disease in elder (Waitzberg & Garla, 2014). DHA is the most abundant long‐chain w‐3 PUFA in the brain (Arterburn, Hall, & Oken, 2006) and accumulates in areas of the brain associated with learning and memory, such as the cerebral cortex and hippocampus (Chung, Chen, & Su, 2008; Gamoh et al., 1999). DHA is incorporated into neuronal membrane glycerophospholipids at the *sn*‐2 position and is involved in regulation of numerous neuronal and glial cell processes, including neurogenesis, synaptogenesis, and neurite outgrowth. Because of its high abundance in the brain, DHA supports membrane protein functions impacting on speed of signal transduction and neurotransmission (Sidhu, Huang, Desai, Kevala, & Kim, 2016). DHA modulates neurotransmitter release, gene expression, membrane‐bound enzyme and ion channel activity, and synaptic plasticity (Horrocks & Farooqui, 2004). Long‐chain ω‐3 PUFAs are considered to be crucial in learning and memory throughout life (Horrocks & Farooqui, 2004; Joffre, Nadjar, Lebbadi, Calon, & Laye, 2014; Luchtman & Song, 2013). n‐3 fatty acids influence the persistence of long‐term memory in rats by maintaining adequate levels of DHA and brain‐derived neurotrophic factor as well as by influencing the activation of NR2B and Fyn during the period of memory formation (Bach et al., 2014). It has been reported that PUFA dietary supplementation in diabetic rats is neuroprotective through an anti‐apoptotic pathway and significantly improves learning and memory abilities (Jia, Heng, Yang, & Gao, 2014). Pérez et al. found that ω‐3 supplementation had two beneficial effects in stressed rats, a strong anti‐stress effect and improved learning (Pérez, Terreros, & Dagnino‐Subiabre, 2013).

### 3.2 Effect of WO on biochemical indices of hippocampal‐dependent cognitive performance

**Figure 2:**
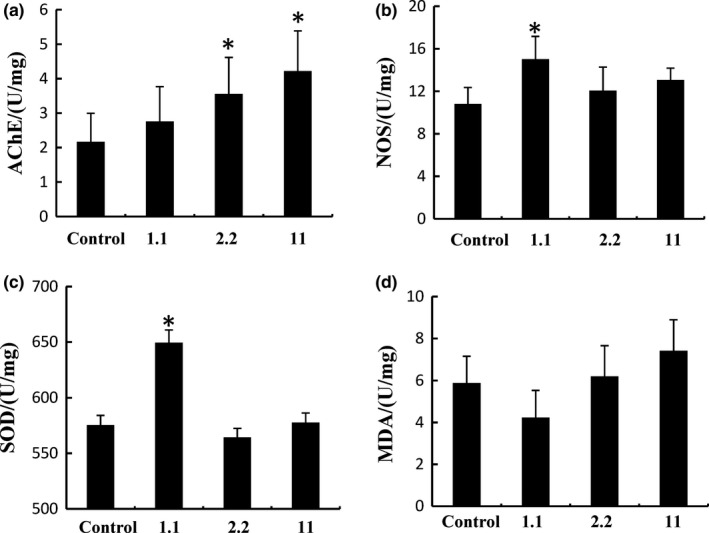
Effect of walnut oil on AChE, NOS, SOD, and MDA levels in rat brain tissue. (a) AChE levels in rat brain tissue. (b) NOS levels in rat brain tissue. (c) SOD levels in rat brain tissue. (d) MDA levels in rat brain tissue. Data are representative of three independent experiments and expressed as mean ±*SD*.*p < 0.05, 1.1  2.2, and 11 g kg^−1^ day^−1^ compared with the control group

Results showed that WO increased AChE activity in a dose‐dependent manner (Figure 2a), while NOS and SOD activities were significantly increased only at 1.1 g/kg (Figure 2b,c). On the other hand, MDA levels were not changed significantly after WO treatment (Figure 2d).** **The activity of AChE, SOD, and NOS is positively associated with hippocampal‐dependent cognitive performance, while MDA, an index of the lipid peroxidation, is negatively associated with such performance (Garthwaite, 2008).

### 3.3 Walnut oil increases the expression of Asic2a and Asic4 in hippocampal neurons

**Figure 3:**
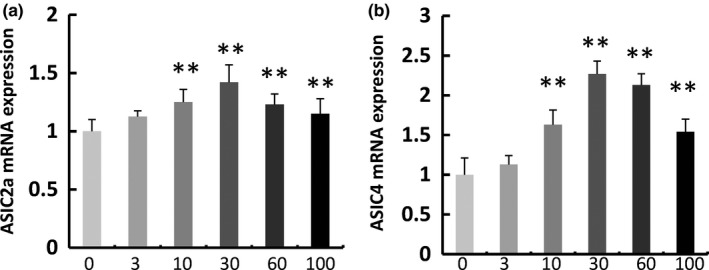
Effect of walnut oil (WO) on the expression of Asic2a and Asic4 in neurons. Cells were incubated with 0, 3, 10, 30, 60, and 100 μg/ml WO for 72 hr, and then, lysate mRNA was subjected to real‐time PCR analysis. Data are representative of three independent experiments and expressed as mean ±*SD*.*p < 0.05, **p < 0.01, compared with the control group

A combination of genetic and pharmacological approaches has revealed the implication of ASIC channels in an increasing number of physiological and pathophysiological processes, most of them associated with extracellular pH fluctuations, including synaptic plasticity, learning, and memory (Du et al., 2014; Wemmie et al., 2002). To evaluate whether treatment with WO improves memory by modulating the expression of *Asic2a* and *Asic4*, we used real‐time PCR to measure the expression levels of mRNA for these genes in cultured hippocampal neurons treated with different doses of WO. When neurons were treated with 10 or 30 μg/ml WO, the expression of *Asic2a* and *Asic4* was increased in a dose‐dependent manner (Figure 3). The expression of *Asic2a and Asic4* reached maximum levels (1.42‐fold and 2.27‐fold higher than control levels, respectively) at a WO concentration of 30 μg/ml. However, the mRNA expression of *Asic2a and Asic4 genes* was reduced in neurons treated with 60 or 100 μg/ml WO compared to the 30 μg/ml group.

**Figure 4:**
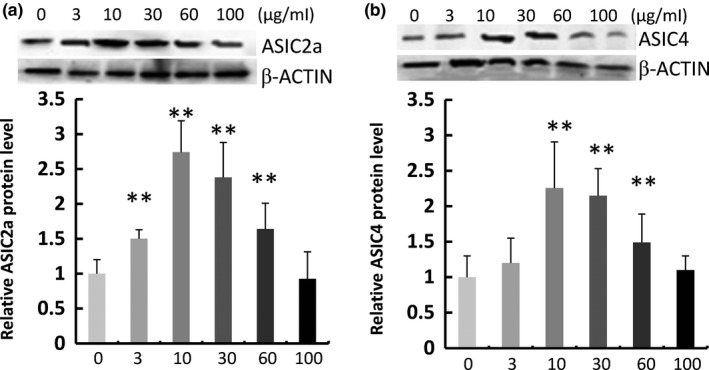
Effect of walnut oil on the expression of ASIC2a and ASIC4 in primary neurons. Western blot analysis shows protein expression in lanes treated with 0, 3, 10, 30, 60, and 100 μg/ml walnut oil for 72 hr. The blots presented are representatives of three independent experiments with similar results in all blots; protein expression was normalized to the expression of the corresponding β‐actin. Data are representative of three independent experiments and expressed as mean ±*SD*, *p < 0.05, **p < 0.01, compared with the control group

These results were confirmed by Western blots (Figure 4). When neurons were treated with WO (3, 10, 30, 60, or 100 μg/ml), the level of ASIC2a protein was increased to 150 ± 13%, 274 ± 45%, 238 ± 50%, 164 ± 37%, and 194.2 ± 20.4% of the control, respectively. When neurons were treated with WO (3, 10, 30, 60, or 100 μg/ml), ASIC4 protein levels were increased to 120 ± 35%, 226 ± 65%, 215 ± 38%, 149 ± 40%, and 110 ± 20% of the control, respectively.

**Figure 5:**
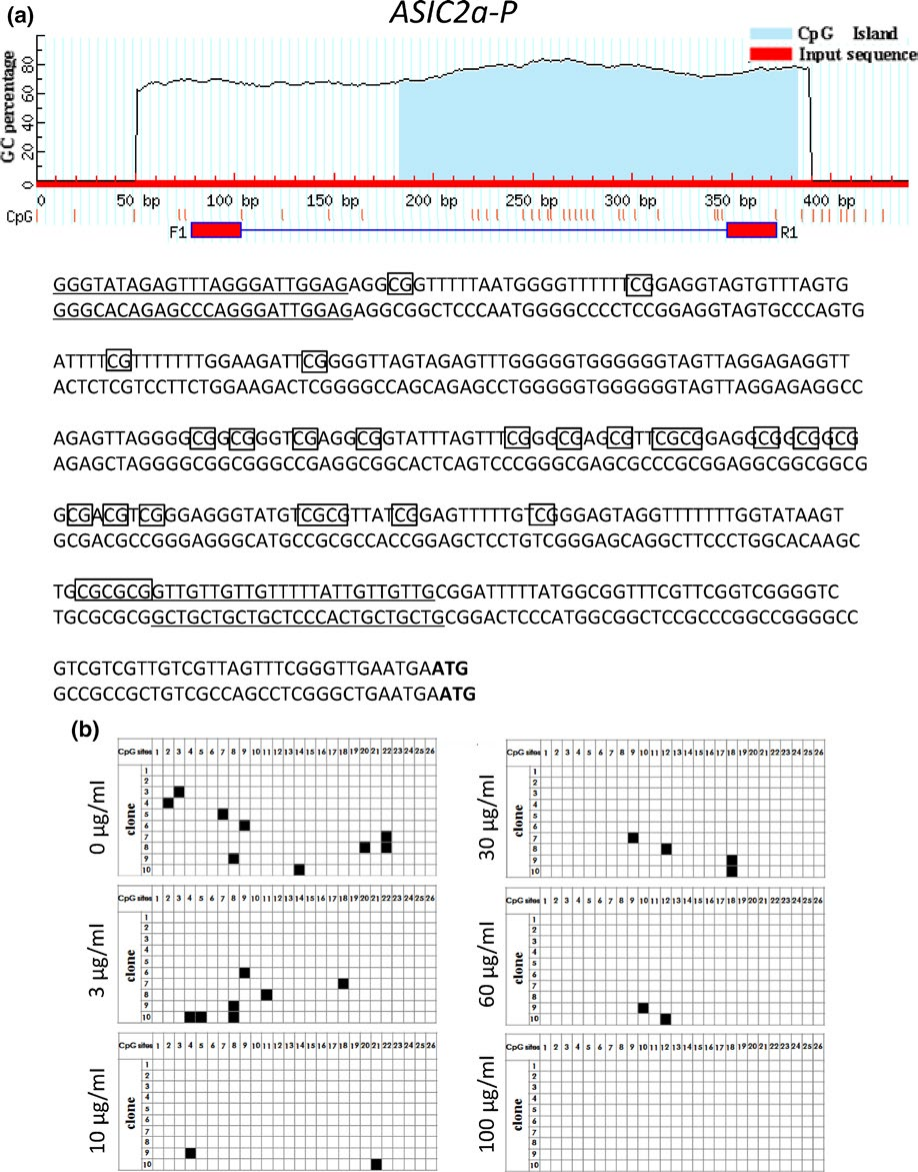
Analysis of gene structure and CpG island of Asic2a. (a) CpG loci of Asic2a‐5′. The red horizontal line represents the input sequence, and the red vertical lines represent the positions of the 26 CpG sites within the 295 bp of Asic2a‐5′. ATG is the start codon of the Asic2a gene. (b) The methylation dynamics of Asic2a‐5′ in primary neurons. Every single row represents a sequenced clone, and each square represents a CpG locus. White and black squares represent unmethylated and methylated cytosine, respectively

**Figure 6:**
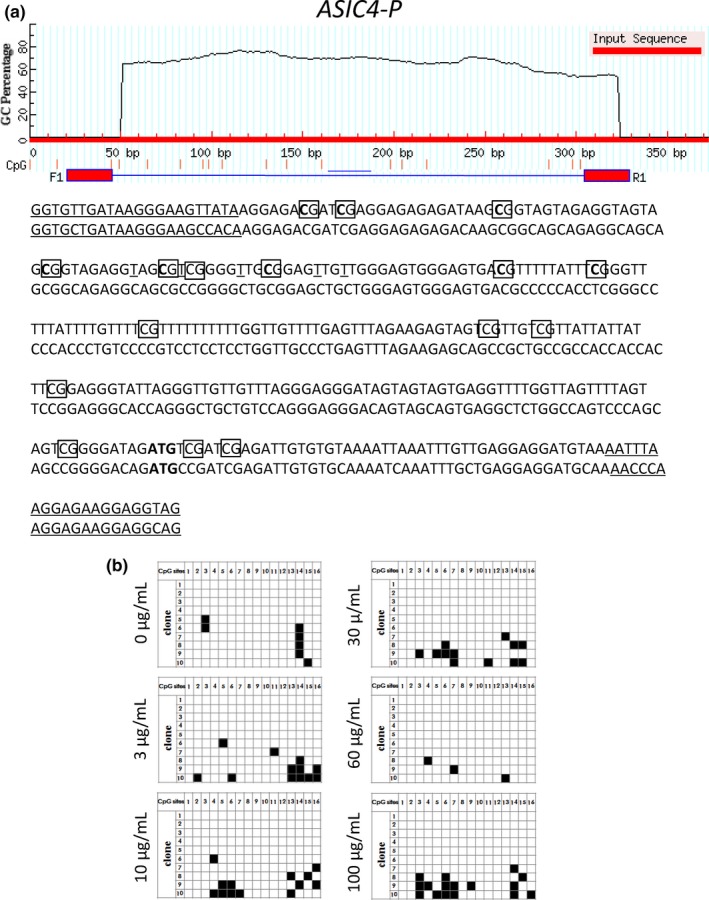
Analysis of gene structure and CpG sites of Asic4. (a) CpG loci of Asic4‐5′. The red horizontal line represents the input sequence, and the red vertical lines represent the positions of the 16 CpG sites within the 347 bp of Asic4‐5′. ATG is the start codon of the Asic4 gene. (b) The methylation dynamics of Asic4‐5′ in primary neurons cells. Every single row represents a sequenced clone, and each square represents a CpG locus. White and black squares represent unmethylated and methylated cytosine, respectively

On the other hand, the methylation status of *Asic2a* and *Asic4* DNA, a possible regulatory mechanism for such genes (Xia et al., 2003), was not changed following WO incubation (Figures 5 and 6).

We conclude that intake of WO might help to prevent cognitive decline. ASIC genes in neurons can be the targets of these compounds contained in the oil. ASICs belong to the amiloride‐sensitive, degenerin/epithelial sodium channel (DEG/ENaC) superfamily. ASICs are voltage‐independent, proton‐gated cationic channels, and their activity has been linked to a variety of physiological and pathological functions in the central and peripheral nervous systems (Pérez et al., 2013). ASICs participate in neuroinflammatory responses, and modulation of these channels could provide a novel therapeutic strategy for controlling neuronal diseases with an inflammatory component (Yu et al., 2015). Accumulating evidence suggests that ASICs represent promising new targets for the treatment of pain and anxiety (Li & Xu, 2015). ASICs contribute to synaptic transmission (Kreple et al., 2014); ASIC1 and ASIC3 isoforms are particularly important in sensory neurons, whereas ASIC1a, alone or in association with ASIC2, is essential in the central nervous system (De & Salen, 2004). ASICs are expressed in both the central and peripheral nervous systems and appear to be associated with many pathophysiological and physiological processes such as hippocampal long‐term potentiation and defects in learning and memory (Lingueglia, Deval, & Lazdunski, 2006). ASICs have been described in many types of neurons throughout the central and peripheral nervous systems (Krishtal, 2003).
